# Isolated schwannoma of the urinary bladder: a case report and review of the literature

**DOI:** 10.11604/pamj.2020.35.108.17745

**Published:** 2020-04-08

**Authors:** Hamid Nasrollahi, Ali Ariafar, Faisal Ahmed, Maral Mokhtari, Ali Eslahi, Mansour Ansari, Umayir Chowdhury

**Affiliations:** 1 Radiation Oncology Department, Shiraz University of Medical Sciences, Shiraz, Iran; 2 Department of Urology, Shiraz University of Medical Sciences, Shiraz, Iran; 3 Urology Research Center, Al-Thora General Hospital, Department of Urology, Ibb University of Medical Sciences, Ibb, Yemen; 4 Pathology Department, Shahid Faghihi Hospital, Shiraz University of Medical Sciences, Shiraz Iran; 5 Radiation Oncology, Breast Diseases Research Center, Shiraz University of Medical Sciences, Shiraz Iran; 6 Student Research Committee, Shiraz University of Medical Sciences, Shiraz, Iran

**Keywords:** Bladder, transurethral resection, schwannoma

## Abstract

The urinary bladder schwannoma is an extremely rare primary urologic tumor. Schwannoma arises from the Schwann's cells in the nerve sheaths and is almost associated with von Recklinghausen's disease. We report a case of schwannoma in a 35-year old female who presented with urinary frequency for 2 months with absence of evidence of von Recklinghausen disease and successfully treated with transurethral resection of bladder lesion (TUR_B). To the best of our knowledge, this represents only the 10^th^ case of benign schwannoma of the urinary bladder in a patient without von Recklinghausen disease. We report a case of an isolated schwannoma of the urinary bladder, and also searched the English literature as we had access to bladder schwannoma.

## Introduction

Peripheral nerve tumors are rare and may arise from the Schwann cells, ganglion cell or capillaries of the nerve cells tumors; among them, schwannoma which is the most common tumor of the peripheral nerves is a benign tumor [1,2]. These tumors may occur intracranially and the most common type of them are acoustic schwannoma [2]. It can occur in every part of the body and the most common location of peripheral schwannoma is lower extremities [3,4]. The majority of bladder cancer cases are transitional cell carcinoma and other less common tumors are squamous cell carcinoma (SCC) and adenocarcinoma [5]. Bladder schwannoma is a rare disease, and herein we report a case of isolated schwannoma of the urinary bladder; we also searched the English literature as we had access to bladder schwannoma.

## Patient and observation

**Figure 1:**
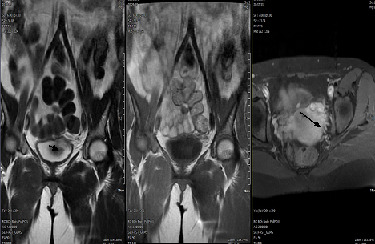
Intramural mass of 7 mm in size located in the upper posterior bladder roof which appears high/low signal on T2/T1W images, uniformly and strongly enhancing on post-contrast images

**Figure 2:**
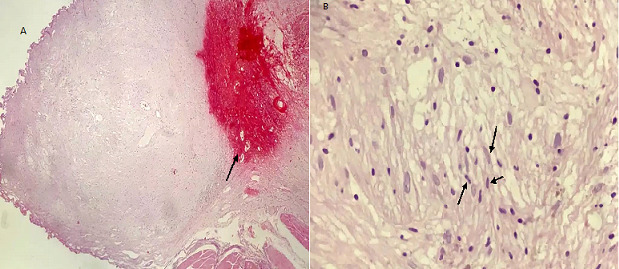
A,B) histopathology slides show a well-defined spindle mass with bland looking nuclei, H&E, X40 and X400

**Figure 3:**
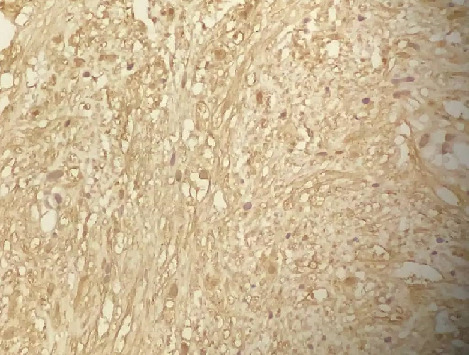
Diffuse S100 immunoreactivity

**Figure 4:**
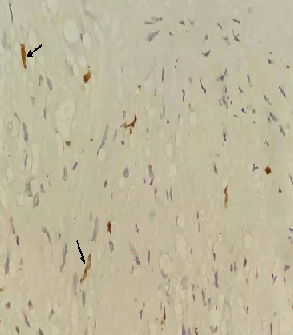
Ki67 immuno-labeling

The patient was a 35-year old female who presented with urinary frequency for 2 months. Her past medical history showed a nonsmoking history and no significant family history. The physical examination was unremarkable. She was treated as urinary tract infection, but there was no improvement in her symptoms. Urine analysis and culture were performed several times that was negative for infection. Other laboratory data were also insignificant. Through a routine evaluation, urinary system ultrasonography (US) revealed a bladder lesion measuring 7 mm. In order to confirm the diagnosis, magnetic resonance imaging (MRI) of the abdomen and pelvic cavity was done; it revealed a lesion in the dome of the bladder (Figure 1). To confirm the diagnosis and for histopathological examination, the patient underwent Transurethral Resection of Bladder Tumor (TURBT) in April 2018. Before starting the procedure, the urethra was dilated till Fr: 28; at first, the tumor location was specified by cystoscopy. Then, it was resected by monopolar cautery from superficial to deeper parts with removal of the muscle layers. Proper bleeders were taken and three way Foley catheter was applied for a continuous irrigation. The patient was transferred to the recovery room with complete stability. The histopathology slides show a well-defined mass composed of bland looking spindle cells. Subsequent immunohistochemistry (IHC) was done which showed diffuse immunoreactivity for S100, but other markers including smooth muscle actin (SMA), desmin, cytokeratin, CD34 and beta catenin were negative. Ki67 was 2-3%, so the diagnosis of schwannoma was confirmed (Figure 2, Figure 3, Figure 4). Then, the neck, abdomen, pelvic and chest computed tomography (CT) scans were done that were normal. Brain and spine MRI and physical examination showed no evidence of other schwannoma or evidence of neurofibromatosis. She had no history of neurofibromatosis in her family. Skin examination was normal and had no café au lait spot. She was not a case of von Recklinghausen disease.

## Discussion

The nerve tumors are named peripheral neural sheath tumors (PNSTs) and it may be benign or malignant. Benign PNST are schwannoma and neurofibroma [4]. Schwannoma which is a component of neurofibromatosis 2 (NF2) may occur incidentally without other criteria of NF [6]. Most of these tumors are sporadic and can occur in both sexes throughout the life and its peak incidence is the 3^rd^ to 6^th^ decades of life [6]. Sporadic schwannoma may occur intracranially or extracranially. The extremities, head, and neck are the most common site of sporadic cases [6,7]. Extracranial lesions may arise from the peripheral nerves, most commonly in the head and neck [7]. Patients usually have complaints that are related to a specific nerve involvement or mass effect. Sometimes, infiltration of adjacent tissues may cause the problem [2]. Treatment of extracranial schwannoma consists of observation or tumor resection, while chemotherapy is not effective in these tumors [2]. Schwannoma of the bladder is extremely rare. Treatment of this type of bladder lesions consists of transurethral or partial resection, and sometimes radiotherapy [3,8]. We found 9 cases of bladder schwannoma in the English literature (Table 1). Most patients (including our case) were females (7:11) with a mean age of 42 years old. Most of them were detected by ultrasonography and the main chief complaints were hematuria, frequency, and pain. Although MRI might be better than CT scan in detecting schwannoma, none can differentiate different types of cancers. The mean tumor size was 2.1 cm. The location of the bladder lesions in 2 cases including our case was in the dome of bladder, and in 1 case it was in the neck of the bladder. Other locations were in the bladder walls. The longest survival was 36 months and no recurrence was reported. None of the patients had received adjuvant treatment [3, 5, 8-14]. It seems that bladder schwannoma is rare with a good prognosis and the best treatment may be surgical tumor removal.

**Table 1:** Characteristics of 10 cases of schwannoma of the bladder

	Age/sex	Presentation	Diagnostic tool	Tumor shape	Size (cm)	Tumor location	Intervention	Survival* (Months)
Srinivasa *et al*. [3]	45/M	Hematuria	US	Polypoid	1.6	Dome of bladder	TURBT	9
Cummings *et al.* [9]	58/F	Pain, Urgency, frequency	US	Cystic lesion	4.5	Lt lateral wall	PC	36
Mosier *et al.* [5]	31/M	Pain, Hematuria	CT	Pedunculated mass	1.7	Lt lateral wall	Surgery	8
Mazdar *et al.* [8]	50/F	Hematuria	US	Solid mass	5.8	Rt lateral wall	TU	5
Bakurov *et al.* [10]	53/M	Hematuria, urgency,	MRI	Solid mass	3.5	Bladder Neck	TU	12
Geol *et al.* [11]	35/M	No symptom	US	Solid mass	3.5	Left lateral wall	PC	12
Gafson *et al.* [12]	52/F	Pain, vomiting, frequency	US	Solid mass	7	Anterior superior wall	Surgery	NA
Ng *et al.* [13]	88/F	Urgency, incontinency	US	Solid mass	20	Left side	No surgery	NA
Jallad *et al.* [14]	25/F	Dyspareunia	NA	NA	NA	NA	NA	NA
Our case	35/F	Frequency	US	Solid	1	Dome of bladder	TURBT	6

**Abbreviations**: TURBT: transurethral resection of bladder tumor; PC: partial cystectomy; US: ultrasonography; M: male; F: female; NA: not available; CT: computed tomography; MRI: magnetic resonance imaging; LT: left; RT: right

* No recurrence detected in all the patients

## Conclusion

Schwannoma of the bladder is rare as a primary urologic tumor and the optimal treatment is surgery with excellent prognosis.

## Competing interests

The authors declare no competing interests.
